# Readmission and death following hospitalization among people with HIV in South Africa

**DOI:** 10.1371/journal.pone.0218902

**Published:** 2019-07-03

**Authors:** Christopher J. Hoffmann, Minja Milovanovic, Cody Cichowitz, Anthony Kinghorn, Neil A. Martinson, Ebrahim Variava

**Affiliations:** 1 Division of Infectious Disease, Johns Hopkins University School of Medicine, Baltimore, Maryland, United States of America; 2 Perinatal HIV Research Unit, Gauteng, South Africa; 3 Johns Hopkins University School of Medicine, Baltimore, Maryland, United States of America; 4 Department of Medicine, Tshepong Hospital, Klerksdorp, South Africa; The Ohio State University, UNITED STATES

## Abstract

**Background:**

Additional approaches are needed to identify and provide targeted interventions to populations at continued risk for HIV-associated mortality. We sought to describe care utilization and mortality following an index hospitalization for people with HIV in South Africa.

**Methods:**

We conducted a prospective cohort study among hospitalized patients admitted to medicine wards at a single hospital serving a large catchment area. Participants were followed to 6 months post-discharge. Hospital records were used to describe overall admission numbers and inpatient mortality. Poisson regression was used to assess for associations between readmission or death and independent variables.

**Results:**

Of 124 enrolled participants, 121 lived to hospital discharge. At the time of discharge the median length of stay of sampled patients was 5.5 days and 105 (87%) participants were referred for follow-up, most within 2 weeks of discharge. By 6 months post-discharge, only 18% of participants had attended the clinic to which they were referred and within the referred timeframe; 64 (53%) had been readmitted at least once and 31 (26%) had died. Self-reported skipping care due to difficulty in access (relative risk 1.3, p = 0.02) and not attending follow-up care on time or at the scheduled clinic or not attending clinic at all (relative risk 1.8 and 2.4, respectively, p = 0.001) were associated with readmission or mortality.

**Conclusions:**

The post-hospital period is a period of medical vulnerability and high mortality. Improving post-hospital retention in care may reduce post-hospital mortality.

## Introduction

Despite the massive scale up of ART in South Africa and important reductions in mortality, HIV-related mortality persists. HIV-associated mortality has declined in South Africa from over 300,000 in 2006 to approximately 110,000 deaths in 2016 [1]. The continued mortality indicates a need to identify pragmatic approaches to identifying and best serving populations at highest risk of dying.

Efforts to reduce mortality may have the greatest impact when focused on populations who are at high risk for dying and are already reached by the health care system. People with HIV (PWH) being admitted to a hospital or with recent hospital discharge are one such population. In South Africa there are approximately 84,633 public sector acute care hospital beds of which approximately 21,769 are adult medicine beds [2] with approximately 360,000 annual HIV admissions [2–5]. Reported inpatient mortality for people with HIV in sub-Saharan Africa ranges from 15–45% [6–8], a level of mortality that may persist following hospital discharge. For example, a study from Tanzania reported 50% mortality one year from hospital discharge [9] and studies from South Africa have reported 18–31% mortality six months from discharge [10, 11]. A better understanding of outcomes, and factors associated with poor outcomes may help to identify opportunities to reduce HIV-associated mortality. The purpose of this study was to estimate post-hospital mortality among PWH admitted to medicine wards at a single hospital in South Africa and to identify factors associated with mortality and readmissions.

## Methods

This prospective cohort study took place at a single tertiary care hospital in the North West province of South Africa. The hospital serves a large urban, peri-urban, and rural catchment area and is part of the only medical care hospital complex in the region. The adult medicine department consists of 216 beds and averages 30 admissions per day with approximately 10,000 annual admissions. HIV testing was routinely provided per the South African Department of Health national HIV testing guidelines [12]. Participants were recruited from adult medicine wards if they met the following criteria: were ≥18 years of age, spoke one of the study languages (English, Setswana, or isiXhosa), were diagnosed with HIV either prior to admission or during the index hospitalization, and were able to complete informed consent or had a legally authorized representative who could perform in person written informed consent. Sequential patients, roomed in open wards, were approached for screening and recruitment. The informed consent criteria led to some of the sickest individuals not being enrolled because they lacked full capacity and often did not have a next of kin present to provide written informed consent. During the period of enrollment the South African ART guidelines stipulated that all people living with HIV with a CD4 count <500 cells/mm^3^ were eligible for ART [13].

This research was conducted according to the principles expressed in the Declaration of Helsinki; written informed consent was obtained from all participants or legally authorized representatives prior to study procedures. The study was approved by the institutional review board of the Johns Hopkins University School of Medicine and the University of the Witwatersrand Human Research Ethics Committee.

### Study procedures

Many of the procedures for this study were similar to those from a prior study that compared care utilization and diagnoses for PWH and people without HIV admitted to the same hospital [14]. Monday through Thursday mornings, trained research staff went to the medical wards and consulted with the nursing staff regarding new admissions of HIV infected patients. Once identified, research staff would approach patients individually, describe the study, and ask each individual if they were willing to participate. Participants who enrolled were given ZAR 20 (US $2) of mobile phone airtime, in keeping with local remuneration rates. An additional ZAR 20 of airtime was provided at each subsequent successful follow-up time point. Due to time constraints the study team was only able to enroll 3–5 patients per day. After administration of informed consent, study staff recorded the participants’ contact information including participant cell phone number, alternative cell phone numbers (household members and/or neighbors) and residential address. This was followed by completion of an enrollment questionnaire, which included demographics, basic past medical history, ART use, care utilization prior to hospitalization, access to medical care, and the chief complaint upon admission. At the time of hospital discharge, the research assistants captured all discharge diagnoses documented in the medical record, medications listed on the participant’s pharmacy card, the results of any CD4 count testing during admission, and the date and location of recommended follow-up care. Participants who died prior to discharge were not included in analyses.

Aggregate numbers of admissions to medical wards and death by HIV-status are maintained by the hospital and were abstracted to determine the total number of potentially eligible patients and the total inpatient mortality rate during the recruitment period.

### Participant follow up

The study team attempted to contact participants 8–12 weeks and 26 weeks after discharge. At each contact point, the study team asked about vital status (if an alternative contact was spoken with) and any appointments attended and/or hospital admissions.

For each follow up contact the study team attempted up to five different phone calls (of all recorded numbers) at different times of the day and on different days of the week (including weekends). If a participant returned to the hospital either for an appointment or was readmitted and the study team was aware of the health care contact, the study team sought to collect any data indicated at that time. Home visits were attempted at 6 months for participants that could not be reached by other means and were not known to have died. At the end of data collection, hospital medical records (that included hospital-based outpatient clinic visit information) were abstracted for all participants to identify attendance at hospital-based clinics, readmission to the index hospital, and recorded death not already identified through other means. In addition, a medical record abstraction was conducted for a participant at the clinic he or she reported attending (any one of the 36 public sector primary care clinics in the catchment area) for a convenience subset of participants to verify self-reported clinic attendance.

### Analysis

Descriptive results are presented as proportions, percentages, and medians with interquartile ranges. Poisson regression with robust variance was used to calculate the incident risk ratio for the dichotomous outcome in approximation of the relative risk of hospital readmission or death for risk factors of interest. These factors included age, sex, CD4 count, timing of HIV diagnosis, length of hospital stay, ART prescription at hospital discharge, reported challenges with care access, care giver at home, and attending care post-hospital. Initially single variable analyses was conducted for each covariate. A multivariable model was calculated using age and sex, selected *a priori*, and all other covariates with a p-value <0.1 in bivariate analysis. All analyses were conducted in STATA, version 14 (Stata Corp. College Station, Texas).

## Results

Between April 6^th^ and July 4^th^ 2016, 222 individuals were screened and 121 participants were enrolled and lived to hospital discharge; three participants died prior to discharge (2.4%). Of the 98 screened patients who were not enrolled, 68 were found to be HIV-negative or not have documented HIV infection, 12 had psychosis or other severe mental illness precluding informed consent, 10 declined participation, 4 were aged <18 years, 3 resided beyond the district in which the hospital was located, and one was aphasic from a stroke. There were approximately 2,274 admissions to medical wards during this period, of which an estimated 1,251 had HIV (thus approximately 10% of all HIV-infected admissions during the study period were enrolled). During this period, 371 medicine ward patients died in the hospital (16% of admissions) of whom 200 (54%) were known HIV-infected, 48 (13%) were known HIV-negative, and 123 (33%) had unknown HIV status.

Among the 121 participants surviving to discharge, the median age was 44 years (interquartile range [IQR] 31, 50), 67 (55%) were women, the median CD4 count during the admission was 260 cells/mm^3^ (IQR 113, 464), and 35% (42/121) reported they “skipped going to hospital or clinic because it is hard to get to” (Table 1). The median length of hospital stay was 5.5 days (IQR 3, 9), 21 (18%) were diagnosed with HIV during the index hospitalization, and 74% had documentation of ART on discharge (others did not meet contemporaneous initiation criteria or were referred to initiate ART post-hospital). Of the 87% (105) scheduled for outpatient follow-up care, 96 (92%) were referred to the specialty clinics at the tertiary care hospital and 9 (8%) were referred to a local public health clinic. Of the follow-up appointments, 51% were scheduled within two weeks of discharge. ART was initiated during the hospital stay for 8 participants not on ART at hospital admission.

**Table 1:** Participant characteristics (n = 121).

	n (%) or median (IQR)
Sex	
Women	67 (55)
Age, median, years	40 (31, 50)
Age, categories, years	
18–35	46 (38)
36–50	45 (37)
>50	30 (25)
CD4 count, median cells/mm^3^	260 (104, 454)
CD4 count group, cells/mm^3^	
≤200	21 (17)
>200	35 (29)
Unknown	65 (54)
HIV diagnosed during hospital stay	21 (17)
Reporting being on ART prior to hospitalization	81 (67)
Duration on ART prior to index hospitalization	
0–2 weeks	16 (13)
2–6 Months	8 (7)
>6 months	57 (47)
Reporting skipping going to clinic or hospital because hard to get to	42 (35)
Reporting having someone to assist with care	101 (83)
Length of stay, median days	6 (3, 9)
Length of stay, days	
≤5	54 (45)
>5	67 (55)
ART status at discharge	
ART initiated during hospitalization	8 (7)
Continued ART from prior to hospitalization	91 (75)
Not documented on ART at discharge	22 (18)
Follow-up scheduled	105 (87)
Timing of follow-up referral (among those with referral)	
≤2 weeks	54 (51)
3–4 weeks	25 (24)
>4weeks	26 (24)
Location of follow-up referral (among those with referral)	
Local clinic	9 (8)
Tertiary hospital specialty clinic	96 (92)

IQR: Interquartile range

The primary diagnoses for the hospitalization ranged across physiologic systems, including neurologic (12/121; 10%); cardiac (7/121; 6%); pulmonary (33/121; 27%) which was further categorized as non-TB pneumonia (16), TB disease (11), and chronic lung disease (6); gastrointestinal (12/121, 10%)–mostly diarrhea; hematologic (5/121; 4%); venous (4/121; 3%); acute kidney disease (2/121; 2%); malignancy (3/121; 2%); and medication toxicity (6; 5%).

By six months post-hospital, all participants had a successful study contact to determine status. Of the 121, 89 (74%) reported, or had a family member report, that they attended a clinic since the index hospital discharge. Of the 89 participants with reported follow-up visits, 46 (52%) visits were to the tertiary hospital specialty clinic and 43 (48%) were to a primary care or community health center closer to their residence. The median time to clinic visit was 5 weeks (IQR: 3, 8); 31 (29%) of the participants with scheduled follow-up went to the clinic within the timeframe scheduled at discharge, but only 19 (18%) post-discharge clinic visits occurred both within the scheduled timeframe and at the clinic to which the participant was referred.

By 6 months post-discharge, 64/121 (53%) participants had a readmission and 31/121 had died (26%); 17 of these deaths occurred during a subsequent hospital admission. During this period 13 participants were readmitted twice and two participants three times. The median time to first readmission was 9 weeks (IQR: 3, 21) and the median time to death was 8 weeks (IQR 3, 12). A cause of death was assigned from hospital medical records for the 17 participants who died during readmission: non-TB pulmonary (6 deaths), TB (2 deaths), cardiovascular (4 deaths), cerebrovascular (1 death), venous thromboembolic disease (2 deaths), and HIV wasting (2 deaths).

Factors associated with the combined death or readmission endpoint were having reported prior to discharge sometimes skipping clinic or hospital due to difficulty with access, longer length of stay, and either not attending the clinic to which they were referred or not attending within the expected time (Table 2). Difficulty getting to clinic and not attending clinic were included in multivariable modeling along with *a priori* selected factors of sex and age. Length of stay lost significance (p>0.1) in multivariable modeling and was dropped. In the multivariable model the relative risk for readmission or death when reporting having sometimes skipped care due to access difficulties was 1.3 (95% confidence interval (CI): 1.0, 1.8) and for lack of clinic follow-up the relative risk, compared to attending the assigned clinic in the assigned time, for visiting any clinic at any time or no clinic at all during follow-up were 1.8 (95% CI: 0.92, 3.6) and 2.4 (95% CI: 1.1, 4.4), respectively (Table 2). As a sensitivity analysis, the multivariable regression was repeated excluding deaths that occurred within two weeks of discharge; this did not change the effect sizes seen in the complete analysis.

**Table 2:** Associations with readmission or death (n = 121).

	Readmit/Died	Relative Risk (95% CI)	Multivariable Relative Risk (95% CI)
Sex			
Women	37 (55)	Referent, p = 0.1	Referent, p = 0.6
Men	37 (68)	1.2 (0.94, 1.6)	1.2 (0.79, 1.7)
Age, categories, years			
17–35	24 (52)	Referent, p_trend_ = 0.2	Referent, p_trend_ = 0.09
36–50	29 (64)	1.2 (0.87, 1.7)	1.2 (0.79, 1.7)
>50	21 (70)	1.3 (0.93, 1.9)	1.4 (0.93, 2.0)
CD4 count			
<200	15 (71)	Referent, p = 0.4	
>200	19 (54)	0.76 (0.5, 1.1)	
Unknown	40 (62)	0.86 (0.62, 1.2)	
HIV diagnosed during hospital stay			
Yes	14 (67)	Referent, p = 0.5	
No	60 (60)	0.9 (0.64, 1.3)	
Reporting skipping going to clinic or hospital because hard to get to			
No	42/79 (53)	Referent, p = 0.008	Referent, p = 0.02
Yes	32/42 (76)	1.4 (1.1, 1.9)	1.3 (1.0, 1.8)
Reporting having someone to assist with care			
No	13/20 (65)	Referent, p = 0.7	
Yes	61/101 (60)	0.93 (0.65, 1.3)	
Length of stay, days			
≤5	27/54 (50)	Referent, p = 0.03	
>5	47/67 (70)	1.4 (1.0, 1.9)	
On ART at discharge, total			
No	15/23 (65)	Referent, p = 0.6	
Yes	59/98 (60)	0.92 (0.66, 1.3)	
Clinic visit on time and at referred clinic			
Yes	6/19 (32)	Referent, p_trend_ = 0.002	Referent, p_trend_ = 0.001
No	42/70 (60)	1.9 (0.95, 3.8)	1.8 (0.92, 3.6)
No clinic attended	26/32 (81)	2.6 (1.3, 5.1)	2.2 (1.1, 4.4)

Clinic file review was completed for 40 participants (45%) who reported clinic visits. Of these reported visits, 87% were able to be verified through review of clinic files when the review was attempted.

## Discussion

This study makes the important contribution of further defining a population with high mortality: PWH surviving to hospital discharge. This study also describes the utilization of care following discharge and the associated morbidity with failure to engage in outpatient care. Among the 121 participants we followed post-hospital, 53% had a readmission and 26% died. Failure to link to recommended care following discharge was common (only 18% attended clinic at the facility and within the time scheduled) and was associated with mortality or readmission.

These findings highlight a population with HIV at particularly high risk of death and add new insight to similar high post-hospital mortality from similar single hospital studies. Studies from South Africa, Tanzania, and Kenya have reported 6 to 12 month mortality ranging between 18 and 50% [5, 9–11, 15].

There are several important limitations to this study. First, this was a single hospital study and the findings may not be generalizable to other care facilities in South Africa. Second, this study sought to use a systematic sampling approach but, because of challenges in enrolling severely ill individuals when a next of kin was not present, it is likely biased toward healthier patients. This potential limitation is further suggested by the observation that only 2.4% of recruited patients died prior to discharge, compared to the 16% overall inpatient mortality. Third, we were unable to capture the difference between scheduled (for ongoing anticipated care or scheduled procedures) and unscheduled readmission. Forth, CD4 count results were only available for 46% of participants. It is possible that with more complete CD4 count data we would have observed an association between CD4 count and post-hospital mortality as has been previously reported [16]. Despite these limitations, this study had several important strengths including 100% participant study follow-up among a population residing in a large catchment area with a single public hospital and verified accuracy of self-reported clinic visits.

This study hospital had already implemented efforts to improve the care transition, including providing all patients with a personal medical record booklet and discharge counseling and care coordination. Here we observed, that despite these efforts, transition in care remained fractured and mortality high during the post-hospital period. Efforts to improve retention along the care continuum, including targeted efforts for the period after hospitalization, may reduce mortality for PWH. Given the cost of both hospital readmission and death, resource intensive interventions may be warranted to address post-hospital morbidity and mortality.

## Supporting information

S1 Dataset (CSV)

## References

[1] UNAIDS. Country: South Africa 2018 [updated 3/18/2018]. http://www.unaids.org/en/regionscountries/countries/southafrica.

[2] Department of Health RoSA. Regulations relating to categories of hospitals. In: Department of Health RoSA, editor. Pretoria: Government of South Africa; 2012.

[3] StatsSA. Mortality and causes of death in South Africa, 2015. In: Africa SS, editor. Pretoria, South Africa: Government of South Africa; 2017.

[4] Roche S, De Vries E. Multimorbidity in a large district hospital: A descriptive cross-sectional study. South African medical journal = Suid-Afrikaanse tydskrif vir geneeskunde. 2017;107(12):1110–5. 10.7196/SAMJ.2017.v107i12.12397 . 29262966

[5] Stuart-Clark H, Vorajee N, Zuma S, Van Niekerk L, Burch V, Raubenheimer P, et al Twelve-month outcomes of patients admitted to the acute general medical service at Groote Schuur Hospital. South African medical journal = Suid-Afrikaanse tydskrif vir geneeskunde. 2012;102(6):549–53. . 22668961 10.7196/samj.5615

[6] Myer L, Smith E, Mayosi BM. Medical inpatient mortality at Groote Schuur Hospital, Cape Town, South Africa, 2002–2009. South African medical journal = Suid-Afrikaanse tydskrif vir geneeskunde. 2012;103(1):28–31. 10.7196/samj.6285 . 23237120

[7] Wajanga BM, Webster LE, Peck RN, Downs JA, Mate K, Smart LR, et al Inpatient mortality of HIV-infected adults in sub-Saharan Africa and possible interventions: a mixed methods review. BMC Health Serv Res. 2014;14:627 10.1186/s12913-014-0627-9 . 25465206 PMC4265398

[8] Akinkuotu A, Roemer E, Richardson A, Namarika DC, Munthali C, Bahling A, et al In-hospital mortality rates and HIV: a medical ward review, Lilongwe, Malawi. Int J STD AIDS. 2011;22(8):465–70. 10.1258/ijsa.2011.011021 . 21795420

[9] Peck RN, Wang RJ, Mtui G, Smart L, Yango M, Elchaki R, et al Linkage to Primary Care and Survival After Hospital Discharge for HIV-Infected Adults in Tanzania: A Prospective Cohort Study. Journal of acquired immune deficiency syndromes. 2016;73(5):522–30. 10.1097/QAI.000000000001107 . 27846069 PMC5129656

[10] Meintjes G, Kerkhoff AD, Burton R, Schutz C, Boulle A, Van Wyk G, et al HIV-Related Medical Admissions to a South African District Hospital Remain Frequent Despite Effective Antiretroviral Therapy Scale-Up. Medicine (Baltimore). 2015;94(50):e2269 10.1097/MD.0000000000002269 . 26683950 PMC5058922

[11] Murphy RA, Sunpath H, Taha B, Kappagoda S, Maphasa KT, Kuritzkes DR, et al Low uptake of antiretroviral therapy after admission with human immunodeficiency virus and tuberculosis in KwaZulu-Natal, South Africa. The international journal of tuberculosis and lung disease: the official journal of the International Union against Tuberculosis and Lung Disease. 2010;14(7):903–8. . 20550776 PMC3207641

[12] Department of Health SA. National HIV counselling and testing policy guidelines. Pretoria: 2015 May 2015. Report No.

[13] South Africa National Department of Health. National Consolidated Guidelines for the Prevention of Mother-to-Child-Transmission of HIV and the Management of HIV in Children, Adolescents, and Adults. In: Department of Health RoSA, editor. Pretoria: Department of Health; 2015.

[14] Cichowitz C, Pellegrino R, Motlhaoleng K, Martinson NA, Variava E, Hoffmann CJ. Hospitalization and post-discharge care in South Africa: A critical event in the continuum of care. PloS one. 2018;13(12):e0208429 10.1371/journal.pone.0208429 . 30543667 PMC6292592

[15] Ousley J, Niyibizi AA, Wanjala S, Vandenbulcke A, Kirubi B, Omwoyo W, et al High Proportions of Patients With Advanced HIV Are Antiretroviral Therapy Experienced: Hospitalization Outcomes From 2 Sub-Saharan African Sites. Clin Infect Dis. 2018;66(suppl_2):S126–S31. 10.1093/cid/ciy103 . 29514239 PMC5850537

[16] Mahlab-Guri K, Asher I, Bezalel-Rosenberg S, Elbirt D, Sthoeger ZM. Hospitalizations of HIV patients in a major Israeli HIV/AIDS center during the years 2000 to 2012. Medicine (Baltimore). 2017;96(18):e6812 10.1097/MD.0000000000006812 . 28471983 PMC5419929

